# Melatonin Act as an Antidepressant via Attenuation of Neuroinflammation by Targeting Sirt1/Nrf2/HO-1 Signaling

**DOI:** 10.3389/fnmol.2020.00096

**Published:** 2020-06-12

**Authors:** Tahir Ali, Qiang Hao, Najeeb Ullah, Shafiq Ur Rahman, Fawad Ali Shah, Kaiwu He, Chengyou Zheng, Weifen Li, Iram Murtaza, Yang Li, Yuhua Jiang, Zhen Tan, Shupeng Li

**Affiliations:** ^1^State Key Laboratory of Oncogenomics, School of Chemical Biology and Biotechnology, Peking University Shenzhen Graduate School, Shenzhen, China; ^2^Institute of Basic Medical Sciences, Khyber Medical University, Peshawar, Pakistan; ^3^Department of Pharmacy, Shaheed Benazir Bhutto University, Sheringal, Pakistan; ^4^Riphah Institute of Pharmaceutical Sciences, Riphah International University, Islamabad, Pakistan; ^5^Signal Transduction Lab, Department of Biochemistry, Faculty of Biological Sciences, Quaid-I-Azam University, Islamabad, Pakistan; ^6^Laboratory of Receptor Research, Shanghai Institute of Materia Medical, Chinese Academy of Sciences, Shanghai, China; ^7^Cancer Centre, The Second Hospital of Shandong University, Jinan, China; ^8^Health Management Center, Shenzhen University General Hospital, Shenzhen University Clinical Medical Academy, Shenzhen, China; ^9^Campbell Research Institute, Centre for Addiction and Mental Health, Toronto, ON, Canada; ^10^Department of Psychiatry, University of Toronto, Toronto, ON, Canada

**Keywords:** melatonin, neuroinflammation, oxidative stress, depression, luzindole

## Abstract

Physical or psychological stress can cause an immunologic imbalance that disturbs the central nervous system followed by neuroinflammation. The association between inflammation and depression has been widely studied in recent years, though the molecular mechanism is still largely unknown. Thus, targeting the signaling pathways that link stress to neuroinflammation might be a useful strategy against depression. The current study investigated the protective effect of melatonin against lipopolysaccharide (LPS)-induced neuroinflammation and depression. Our results showed that LPS treatment significantly induced depressive-like behavior in mice. Moreover, LPS-treatment enhanced oxidative stress, pro-inflammatory cytokines including TNFα, IL-6, and IL-1β, NF-κB phosphorylation, and glial cell activation markers including GFAP and Iba-1 in the brain of mice. Melatonin treatment significantly abolished the effect of LPS, as indicated by improved depressive-like behaviors, reduced cytokines level, reduced oxidative stress, and normalized LPS-altered Sirt1, Nrf2, and HO-1 expression. However, the melatonin protective effects were reduced after luzindole administration. Collectively, it is concluded that melatonin receptor-dependently protects against LPS-induced depressive-like behaviors via counteracting LPS-induced neuroinflammation.

## Introduction

Major depressive disorder (MDD) is a major health concern associated with brain and immune system abnormalities. It plays a significant role in the global burden of diseases by affecting people in all communities across the world. According to recent epidemiological surveys, it has been estimated that by 2020, MDD will be the 2nd leading diseases worldwide (Kessler et al., 2003; Kessler and Bromet, 2013). The central nervous system (CNS) responds to the pathogen and several cellulars stress processes through neuro-inflammation, which can induce molecular dysregulation and can be a critical control point for the development of depression (Singhal et al., 2014; Tohidpour et al., 2017). It has been reported that failure in adaptation to psychological or physical stress can lead to depression which is mediated by an inflammatory response and cytokines (Wu et al., 2012; Jeon and Kim, 2016). Several lines of evidence from both clinical and experimental data have strongly proposed that internal and external stress significantly affects the expression of depressive symptoms and their persistence is associated with immunological abnormalities (Wu et al., 2012; Hsieh and Yang, 2013; Jeon and Kim, 2016). An elevated level of microglia activation has been reported in individuals with depression who commit suicide, suggesting that neuroinflammation contributes a significant role in the pathogenesis of depression (Brites and Fernandes, 2015). The immune system could also affect the CNS through cytokines, which not merely participate in cell-to-cell communication but also affect the regulatory and processing mechanism of neurochemicals and neuroendocrine that are the key regulator of physiological and behavioral alterations (Jeon and Kim, 2016). Activated peripheral immune system increase cytokines production and flow to CNS, where they induce the activation of astrocytes and microglia, which in turn elevates cytokines production (Muller and Ackenheil, 1998; Song and Wang, 2011). Moreover, the crosstalk between microglia and astrocytes under stress condition involve in neuroinflammation that leads to dysfunction in the neurotrophic system, which may contribute to the pathogenesis of MDD (Song and Wang, 2011).

Dysregulation in the redox-sensitive signalings has been shown to play a major role in an immune imbalance that leads to depression (Martin-de-Saavedra et al., 2013). Enhanced reactive oxygen species (ROS) overwhelms the antioxidant defense system that leads to oxidative stress, which in turn participates in the pathogenesis of numerous diseases including neurological abnormalities (Alfadda and Sallam, 2012; Salim, 2014; Song et al., 2018). Accumulative results support the increased expression of pro-inflammatory cytokines that occurred via the activation of transcription factor NF-κB under stress condition (Munhoz et al., 2008; Kassan et al., 2013; Jeon and Kim, 2016), as demonstrated by NF-κB activation by inflammatory cytokines and lipopolysaccharide (LPS; Rushworth et al., 2005; Kassan et al., 2013). Apart from its pro-inflammatory function, NF-κB is also involved in the oxidative/anti-oxidative stress regulation, which in turn affect cytokine production (Kratsovnik et al., 2005; Kawai and Akira, 2007; Lugrin et al., 2014; Djordjevic et al., 2015). Interestingly, NF-κB regulates Nrf2 transcription and activity, which not only elevates antioxidant capacity but also induces the expression of a neuroprotective protein, such as brain-derived neurotrophic factors (BDNF), anti-inflammatory protein Hemoxygenase-1 (HO-1), and anti-inflammatory cytokines (Sekio and Seki, 2015; Cuadrado et al., 2018), indicating the intricate interplay between neuroinflammation and oxidative stress systems. Further, Sirt1 regulation has been also currently reported in mood disorders both in the animal models as well as humans (Kishi et al., 2010; Iacono et al., 2015; Luo and Zhang, 2016). Numerous studies have reported that Sirt1 can reduce inflammation as well as oxidative stress (Alcendor et al., 2007; Salminen et al., 2008; Iacono et al., 2015), therefore, a stress inducer like LPS may suppress Sirt1 expression (Hurley et al., 2014; Ge et al., 2015; Ali H. et al., 2015; Shah et al., 2017).

Numerous studies have reported the beneficial effects of exogenous melatonin on the brain, which might include the activation of melatonin membrane receptors (MMRs). Luzindole (*N*-acetyl-2-benzyltryptamine) is a well-known high selective MMR antagonist and is widely employed to study the action of melatonin on the signaling pathways and the associated neuroendocrine and functional responses (Ortiz-López et al., 2016; Estaras et al., 2019).

Melatonin (N-acetyl-5-methoxytryptamine) is the main neurohormone of pineal glands. It regulates the major physiological process via its receptors (MT1, MT2), which are specific G-protein coupled receptors to regulate downstream molecules phospholipase C (PLC), guanylyl cyclase (GC), adenylyl cyclase (AC), cyclic guanosine monophosphate (cGMP), and as well as calcium and potassium channels (Negi et al., 2011; Guijarro-Munoz et al., 2014; Taniguti et al., 2018). MT1 and MT2 receptors are expressed in the CNS including the hippocampus, suprachiasmatic nucleus (SCN), and tegmental areas together as well as separately (Hirsch-Rodriguez et al., 2007). Melatonin is a key immunomodulatory and neuroprotective via its antioxidative and anti-inflammatory mechanisms, presumably by encountering the free radicals (Hirsch-Rodriguez et al., 2007; Guijarro-Munoz et al., 2014; Cecon et al., 2018; Hardeland, 2018). Previous studies reported that the free radical scavenging actions of melatonin are receptor-independent (Rehman et al., 2019; Zhao D. et al., 2019; Maher et al., 2020; Zhi S. M. et al., 2020), while it also regulates antioxidant enzymes including superoxide dismutase and catalase through receptor-dependent mechanisms (Reiter et al., 2003; Rodriguez et al., 2004; Reiter et al., 2016; Zhao et al., 2018). Herein we tried to explore the molecular mechanisms of melatonin involved in stress-induced neuroinflammation, an essential strategy against MDD.

## Materials and Methods

### Animal and Drug Treatment

**FIGURE 1:**
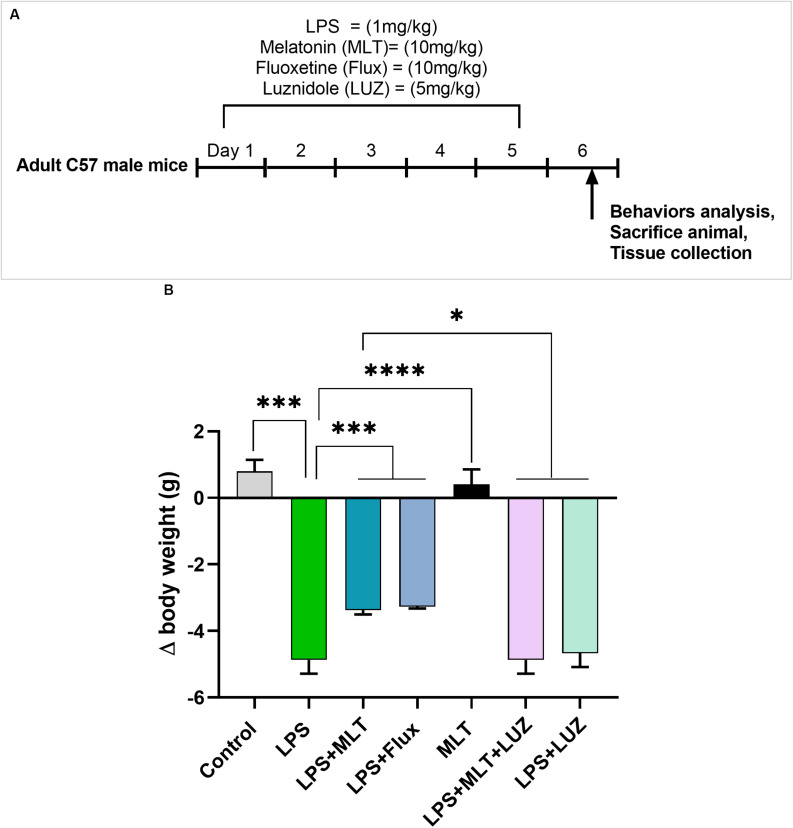
Drug treatment schedule and LPS effect on body weights **(A)** Drugs treatment schedule. Melatonin/Fluoxetine/Luzindole was administrated (i.p) 1 h before the LPS treatment for 5 days. **(B)** Relative body weights differences. (*n* = 6 per group). Data are expressed as mean ± SEM, andresults were analyzed using one-way ANOVA followed by *post-hoc* analysis. *p* < 0.05 wasconsidered statistically significant. *p < 0.05, **p < 0.01, ***p < 0.001, ****p < 0.0001.

Adult C57BL/6J male mice weighing 25–30 *g* (7–8 weeks) were purchased from Guangdong medical laboratory animal center, China. The experimental animals were housed at Laboratory Animal Research Center, Peking University Shenzhen Graduate School, under 12 h light/12 h dark cycle at 18–22°C, and had free access to diet and tap water throughout the study. The experimental procedures were set in such a way to minimize mice suffering. All experimental procedures were carried out according to the protocols approved by the Institutional Animal Care and Use Committee of Peking University Shenzhen Graduate School. The experimental animals were divided into seven groups (each group *n* = 6): normal saline-treated, LPS (1 mg/kg/day) treated, LPS + Melatonin (10 mg/kg/day) treated, LPS + Fluoxetine (10 mg/kg/day), Melatonin (10 mg/kg/day) treated, LPS + Melatonin + luzindole (5 mg/kg/day) treated, and LPS + luzindole treated. Drugs (Melatonin Fluoxetine, and luzindole) were treated intraperitoneally (12 pm to 2 pm) 1 h before LPS treatment daily for 5 days. Both drugs melatonin and luzinolde were dissolved in 5% DMSO and were administrated according to the previously described protocol (Moezi et al., 2011; Zieliñska et al., 2016; Wang et al., 2017). The drug treatment schedule has been shown in Figure 1A. After 24 h of last LPS injection, mice were sacrificed. Serum and brain tissues were collected and stored at freezing temperature (−80°C) until further analysis.

### Open Field Test

Open field test (OFT) was performed according to the previously developed protocols (Zhao X. et al., 2019). Briefly, mice were adapted to the experimental room for 1 h and were placed in the chamber of 45 × 45 × 30 cm. A total of 15 min video was recorded to observed the mice locomotor activity. The total distance covered by mice was measured, analyzed, and expressed in meters.

### Sucrose Preference Test

A sucrose preference test (SPT) was performed (Couch et al., 2016) while using a two-bottle free-choice paradigm. Mice were habituated with a 1% sucrose solution for 3 days and finally grouped randomly. To assess THE individual sucrose intake, mice were deprived of water and food for 24 h on the 3 days of drug administration. On the next day, each mouse had free access to two bottles containing sucrose and water, respectively. The position of water and sucrose-containing bottles were changed after 12 h. Finally, the volume of consumed water and sucrose solution were recorded and calculated by the following formula:

$$\text{SPT}=\frac{\text{Sucrose}\,\text{consumption}}{\text{water}\,\text{and}\,\text{Sucrose}\,\text{consumption}}\times 100\%$$

### Forced Swimming Test

The forced swimming test (FST) was performed according to previously developed protocols (Sekio and Seki, 2015). The experimental animals were trained for swimming and pre-experiment FST was performed to select healthy and normal mice. To perform the FST, the animals were placed in a Plexiglas cylinder (height: 70 cm, diameter: 30 cm) filled with water over the 30 cm level at a temperature of 23 ± 1°C. The video was taped for 6 min and the last 5 min were blindly analyzed. Mice were considered immobile when they remained floating motionless in the water and just making a move to keep their nose above the water surface. The horizontal movement of the animals throughout the cylinder was defined as swimming while vertical movement against the wall of the cylinder was defined as climbing. EthoVision XT was used to record the video and analysis.

### Tail Suspension Test

Tail suspension test (TST) was performed according to the previously described protocol (Steru et al., 1985; Zhao X. et al., 2019). The experimental animals were suspended upside down by tails 40 cm above the floor by adhesive tape placed 1 cm from the tail tip. The immobility time was scored for the first 2 min of a total 4 min video. EthoVision XT software was used for TST video recording and analysis.

### Serum ROS Level Measurement

Reactive oxygen species were analyzed by a previously developed method (Hayashi et al., 2007; Ali et al., 2019). Briefly, hydrogen peroxide/serum (5 μL/well) was added to 140 μL of 0.1 M sodium acetate buffer (pH 4.8) in a 96-well microtiter plate. A mixture (100 μL) which was prepared from reagent R1 (100 μg/ml DEPPD in 0.1 M Sodium acetate buffer, pH 4.8) and R2 (4.37 μM ferrous sulfate in 0.1 M sodium acetate buffer) at a ratio of 1:25 was added in each well. Then, after free incubation of 1 min, absorbance at 505 nm was measured using a plate reader (Envision 2104, PerkinElmer).

### TBARs Assay

Thiobarbituric acid reactive substance (TBARs) level was estimated (Ali T. et al., 2015) to determine the damage to lipids caused by ROS in various experimental groups. Briefly, 0.1 ml of sample (Hippocampal tissue immunomodulatory), 0.1 ml FeSO4, 0.1 ml Tris–HCl, 0.6 ml distilled water, and 0.1 ml Ascorbic Acid were incubated at 37°C in a test tube for 15 min and then 1 ml TCA and 2 ml TBA were added. These plugged test tubes were incubated for 15 min at 100°C followed by centrifugation at 3000 rpm for 10 min. The supernatant O.D. was determined at 532 nm and the following formula was applied to estimate TBARs as nM/mg protein: TBARs (nM/mg protein) = O.D × Total volume × Sample volume × 1.56 × 105 × mg protein/ml (1.56 × 105 = Molar Extinction Coefficient).

### ELISA

The frozen hippocampal tissue was lysed with RIPA buffer and homogenized on ice. Supernatants were collected after centrifugation at 10,000 *g* for 10 min and stored at freezing temperature for further analysis. The expression of cytokines was quantified using enzyme-linked immunosorbent assay (ELISA) kits according to the manufacturer’s protocols (IL-6 Cat NO: RK00008, IL-1β Cat NO: RK00006, and TNFα Cat NO: RK00027, ABclonal Biotechnology Co., Ltd, Wuhan, Hubei Province, China). Briefly, after washing the wells of 96-well plate, 100 μL standard/sample (sample serum/hippocampus tissues) was added and incubated for 2 h at 37°C. The plate was then washed and a biotin-conjugated antibody (1:30) was added to each well. The plate was incubated for 1 h at 37°C. streptavidin-HRP was added for 30 min at 37°C. Finally, the reaction was stopped and the optical density was measured accordingly.

### Immunofluorescence

Immunofluorescence staining was performed according to previously reported protocols (Shah et al., 2017). Briefly, brain tissue sections (20 μm thick) were washed with PBS for 15 min (5 min × 3). After washing, the sections were treated with blocking buffer (10% Goat serum in 0.3% Triton X–100 in PBS) for 1 h at room temperature. After blocking the tissue was treated with primary antibodies with a dilution ratio of 1:500 μL (Iba1, GFAP) for overnight at 4°C. Next day secondary antibodies 1:400 μL (Alexa Flour secondary antibodies, ThermoFisher, Waltham, MA, United States) were applied at room temperature for 1 h. The sections were washed with PBS for 5 min three times. After washing, sections were transferred to slides, and glass coverslips were mounted using the mounting medium. The images were taken under inverted fluorescence microscope IX73 Olympus. ImageJ software was used to quantify the relatively integrated density of GFAP and Iba-1.

### Western Blotting

To extract the protein hippocampal tissue was lysed with RIPA buffer and homogenized on ice. Supernatants were collected after centrifugation (10,000 rpm for 10 min). The immunoblotting was also performed according to the developed protocols. Briefly, denatured samples (boiled at 100°C for 5 min) were separated on SDS-PAGE and then transferred to the nitrocellulose membrane. The membrane was blocked in with non-fat milk in TBST (Tris–buffered saline, 0.1% Tween 20), then incubated in primary antibody (1:1000 dilution used for Nrf2, *p*-NFkB, NFkB, *p*-GSK-3b, GSK-3b, Ho-1, GAPDH, Tubulin, 1:2000 dilution was used for *p*-Akt, and Akt) overnight at 4°C. The primary antibodies were diluted according to the company provided protocol. The next day membrane was treated with a secondary antibody (1:1000) for 1 hr at 4°C. For detection, the ECL Super signal chemiluminescence kit was used according to the manufacturer’s protocol. Blots were developed using Chemidoc mp Bio-red, Hercules, CA, United States. The densitometry analysis of the bands was performed using image lab software.

### List of Antibodies Used

Anti-Nrf2 (cell signaling, Lot: 12721), Anti-p-NFkB (Cell signaling, Lot: 3033), Anti-NF-κB (Cell signaling, Lot: 8242), Anti-p-GSK3b (Santa Cruz, Lot: sc-11757), Anti-GSK3B (Santa Cruz Lot: sc-9166), Anti-p-Akt (Cell signaling, Lot: 4060), Anti-Akt (Cell signaling, Lot: 4691), Anti-HO-1 (Cell signaling, Lot: 70081), Anti-Sirt1 (Cell signaling, Lot: 8469), Anti-GAPDH (Cell signaling, Lot:5174), and Anti-Tubulin (Santa Cruz, Lot: sc-8035).

### Statistical Analysis

Western blot bands and morphological data were analyzed using ImageJ software (Image J 1.30) and analyzed by SPSS Statistics 21 (IBM, United States) and GraphPad Prism 5 software. Data were presented as mean ± SEM. One way/two way ANOVA followed by *post hoc* Tukey/Bonferroni Multiple Comparison tests were performed to compare different groups, using the graph-pad prism-5 software. *P* < 0.05 was regarded as significant. (^∗^): *p* < 0.05, (^∗∗^): *p* < 0.01), and (^∗∗∗^): *p* < 0.001.

## Results

### Melatonin Reduced LPS Induced Depressive-Like Behavior

**FIGURE 2:**
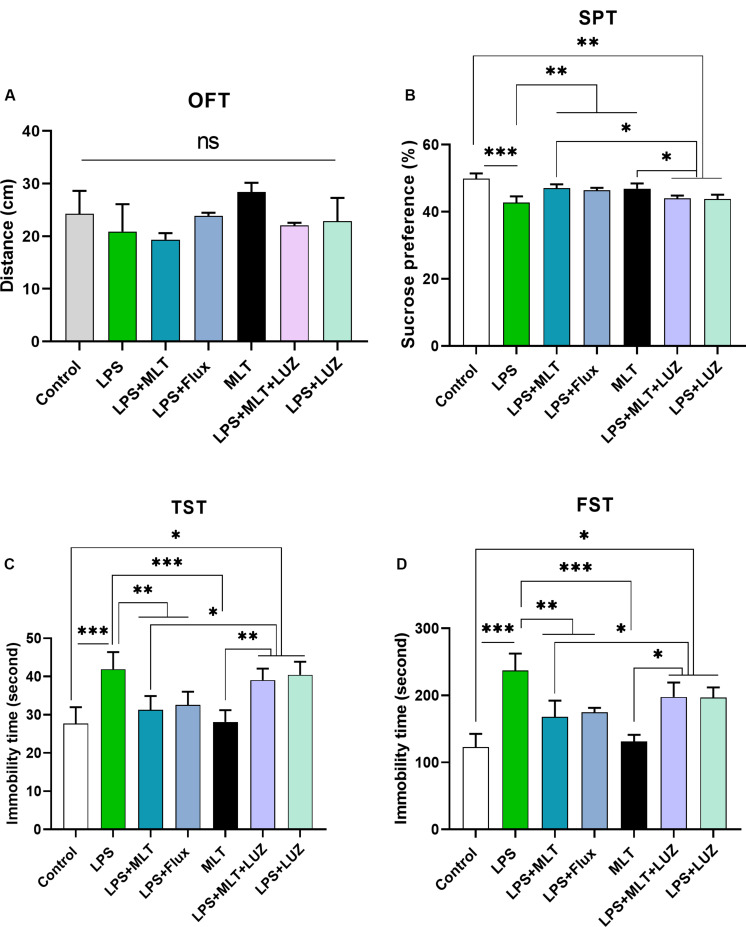
Melatonin ameliorated depressive-like behavior induced by LPS. **(A)** Locomotor activity analysis by open field test, **(B)** Sucrose preference test (SPT), **(C,D)** immobility analysis by tail suspension and FST. (*n* = 6 per group). Data are expressed as mean ± SEM, and results were analyzed using one-way ANOVA followed by *post-hoc* analysis. *p* < 0.05 was considered statistically significant. *p < 0.05, **p < 0.01, ***p < 0.001.

Previous studies have reported that LPS can induce depressive-like behaviors (O’Connor et al., 2009; Arioz et al., 2019). Herein, LPS induced depressive-like behaviors were measured at 24 h post LPS (last) injection by assessing changes in the body weight (Figure 1B), locomotor activity (Figure 2A), and immobility duration. LPS-treated mice showed sucrose preference less than 65% for a 1% sucrose solution (Figure 2B) and increased immobility duration (Figures 2C,D), however, this effect was blocked by pre-melatonin treatment. Moreover, it was interesting in the report that luzindole (melatonin receptor inhibitor) treatment significantly abolished melatonin protective effects, suggesting that endogenous melatonin can block the onset of LPS induced depressive-like behaviors. Fluoxetine was used as a positive control as reported previously (Todorovic and Filipovic, 2017; Micheli et al., 2018).

### Melatonin Modulated LPS-Induced Oxidative Stress and Altered AKT/GSK3β Signaling

**FIGURE 3:**
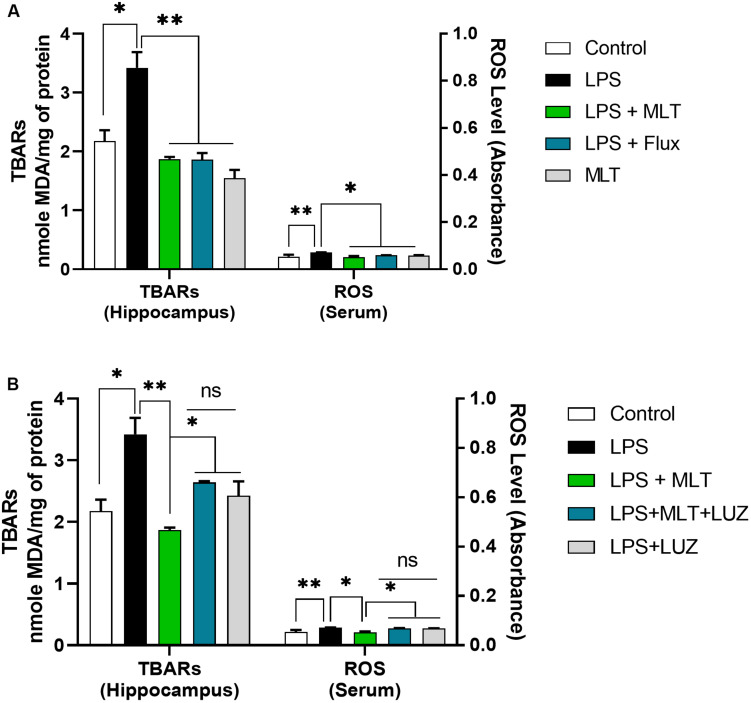
Melatonin attenuated oxidative stress. **(A,B)** Column graphs representing the quantified ROS levels in serum and TBARs level in the hippocampal tissues of experimental mice. Data are expressed as mean ± SEM, and results were analyzed using one-way ANOVA followed by *post-hoc* analysis. *p* < 0.05 was considered statistically significant. *p < 0.05, **p < 0.01.

**FIGURE 4:**
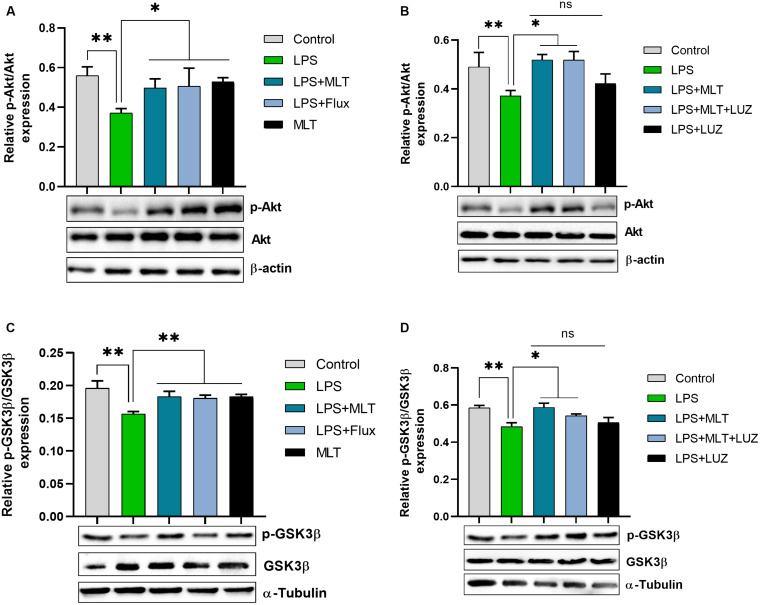
Melatonin decreased Akt and GSK3β phosphorylation induced by LPS. **(A)** Relative Phospho-Akt expression normalized by total Akt. **(B)** Represent Akt Phosphorylation in the hippocampus area of experimental animals normalized by total Akt. **(C,D)** Column graphs representing relative p-GSK3β expression in the hippocampus of animal models treated with LPS/drugs normalized by total GSK. Data are expressed as mean ± SEM, and results were analyzed using one-way ANOVA followed by *post-hoc* analysis. *p* < 0.05 was considered statistically significant. *p < 0.05, **p < 0.01.

An array of studies from experimental (*in vitro* and *in vivo*), as well as human studies, support the role of oxidative stress in the progression of diseases including neurological disorders (Kovacic and Somanathan, 2012; Hsieh and Yang, 2013). Moreover, altered redox-sensitive signaling including Akt/GSK3b accelerates free radical generation, followed by cytokines production, which can lead to neuroinflammation (Weichhart and Säemann, 2008; Kim et al., 2009; Manning and Toker, 2017). Herein, our results indicated elevated serum ROS (Figure 3A), hippocampus TBARs (Figure 3B), and Akt/GSK3b phosphorylation (Figures 4A,B) in the LPS-treated mice, which were significantly reversed by melatonin treatment. However, after luzindole treatment, the effects of melatonin were abolished (Figures 4C,D), suggesting the anti-oxidative capacity of melatonin.

### Melatonin Reduced Neuro-Inflammation Elicited by LPS

**FIGURE 5:**
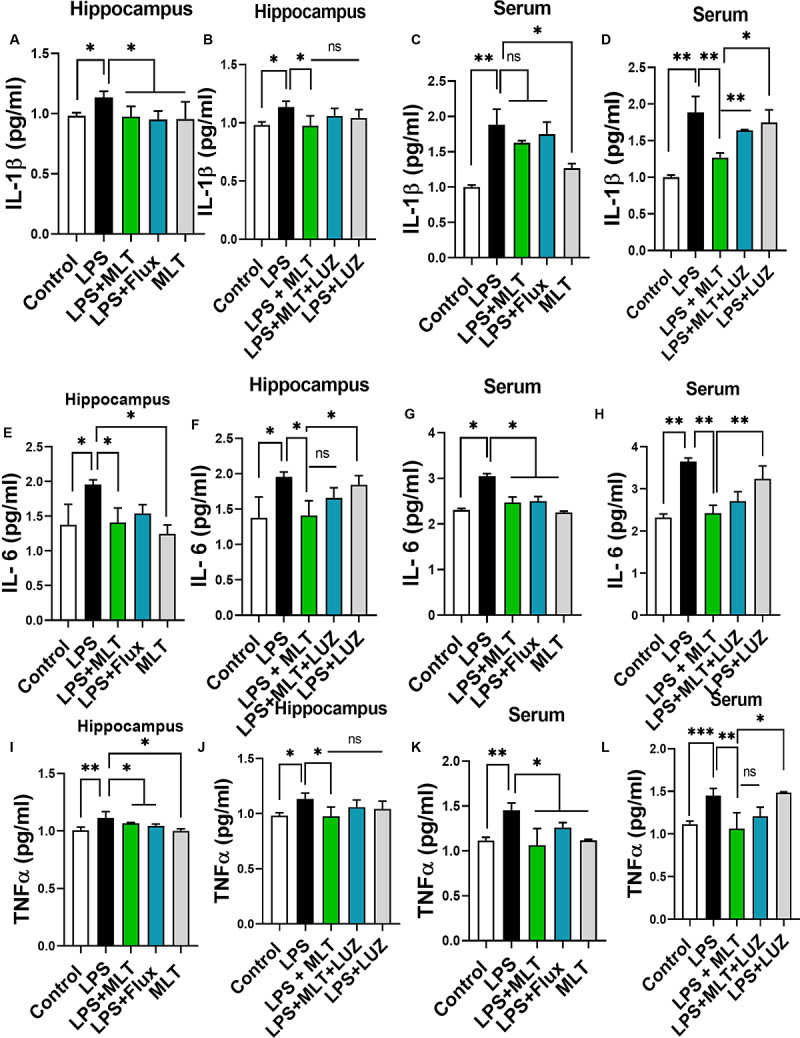
Melatonin attenuated LPS-induced Cytokines elevation. **(A,B)** IL-1β level in the hippocampus, **(C,D)** Serum IL-1β level, **(E,F)** IL-6 level in the hippocampus, **(G,H)** Serum IL-6 level, **(I,J)** TNFα level in the hippocampus, and **(K,L)** Serum TNFα level. Data are expressed as mean ± SEM, and results were analyzed using one-way ANOVA followed by *post hoc* analysis. *p* < 0.05 was considered statistically significant. *p < 0.05, **p < 0.01, ***p < 0.001.

LPS is a well-known inflammatory agent and it activates astrocytes and microglia followed by the pro-inflammatory cytokine-like TNF-α and IL-6, productions (Rushworth et al., 2005; Velasquez and Rappaport, 2016; Song et al., 2018). Both serum and tissue ELISA results indicated that LPS-treatment significantly accelerated pro-inflammatory cytokines including IL-1β (Figures 5A,C), IL-6 (Figures 5E,G), and TNF-α (Figures 5I,K) concentration while melatonin treatment reversed these changes.

**FIGURE 6:**
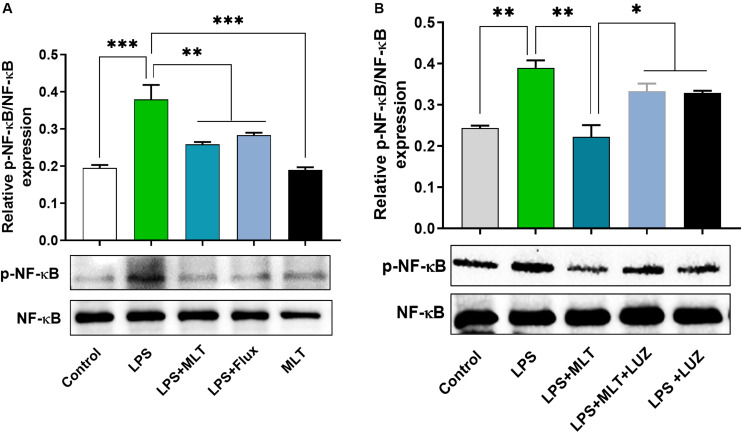
Melatonin reduced LPS-induced NF-kB expression. **(A,B)** Represents immunoblots indicating the expression of *p*-NF-κB/NF-κB in the hippocampus of experimental mice. All the values are expressed as mean ± SEM: ANOVA followed by *post hoc* analysis. *p < 0.05, **p < 0.01, ***p < 0.001.

**FIGURE 7:**
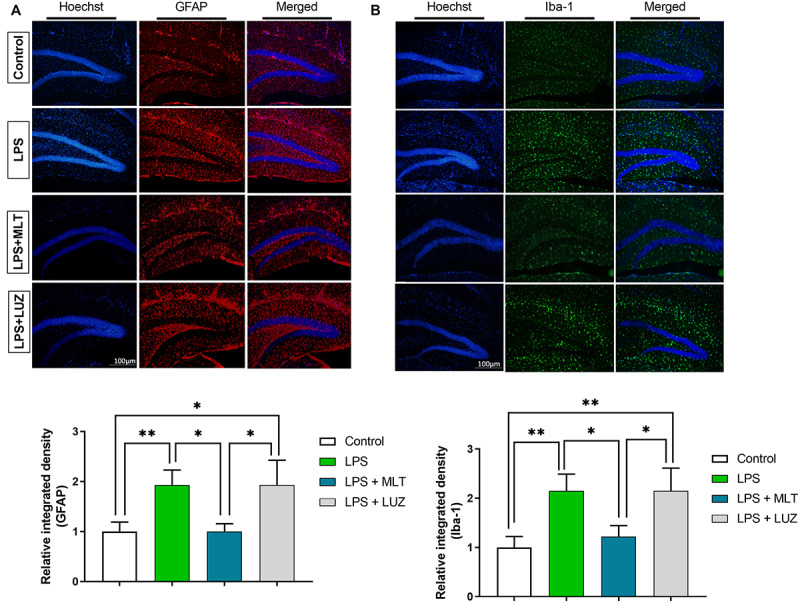
Melatonin inhibited microglia and astrocyte activation by LPS. **(A)** Iba-1 positive microglia (green) and **(B)** GFAP-positive astrocytes (red). Column graphs representing the immunoreactive intensity of the microglia as well as astrocytes. *p* < 0.05 was considered statistically significant. *p < 0.05, **p < 0.01.

Moreover, numerous stimuli including cytokines, chemokines, LPS, and oxidative stress induce NF-κB activation, which subsequently plays a key role in the neuroinflammation by accelerating cytokines productions (Mémet, 2006; Kawai and Akira, 2007). Herein, our results showed markedly increased NF-κB phosphorylation (Figure 6A) in the brain of LPS-treated mice, which was normalized after melatonin treatment. Furthermore, melatonin treatment attenuated LPS-mediated glial cells activation markers including GFAP and Iba-1 expression (Figures 7A,B). However, after the melatonin receptor blocking by luzindole melatonin protective effect against LPS induced neuroinflammation was reduced (Figures 5B,D,F,H,J,L, 6B, 7), suggesting the key protective potential of melatonin against LPS induced neuroinflammation.

### Melatonin Regulates Nrf2/Sirt1/HO-1 Expression in the Hippocampus Area of Experimental Animals

**FIGURE 8:**
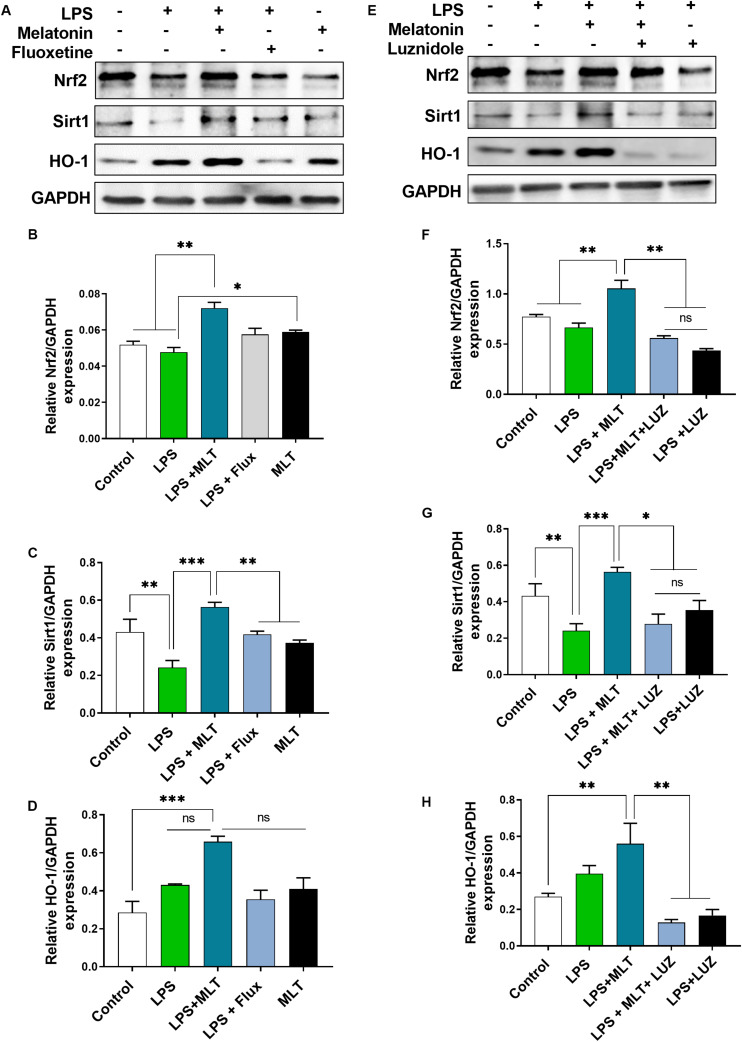
Melatonin elevated Nrf2, Sirt1, and HO-1 expression receptors dependently in the hippocampus area. **(A,E)** Representative blots show Nrf2, Sirt1, and HO-1 expression, **(B,F)** representing Nrf2 relative expression, and **(C,G)** shows Sirt1 expression in the hippocampus of the animal model. **(D,H)** Quantitative analysis of HO-1. Quantified results were normalized to GAPDH. All the values are expressed as mean ± SEM: ANOVA followed by *post hoc* analysis. *p < 0.05, **p < 0.01, ***p < 0.001.

Nrf2 is a well-known master regulator of redox homeostasis and cytoprotective protein involved in antioxidant reactions as well as inflammation (Djordjevic et al., 2015; Luo et al., 2018) while Sirt 1 has been currently implicated in depression (Abe et al., 2011; Libert et al., 2011; Chung et al., 2013). In the present study, our results demonstrated that LPS-treatment had no significant effect on Nrf2 expression in the hippocampal area of the brain. However, increased Nrf2 expression was detected in the brain of the melatonin-treated mice (Figures 8A,B). Similarly, melatonin-treatment enhanced Sirt1 expression in the brain of mice, which was suppressed by LPS treatment (Figures 8A,C). Interestingly, luzindole treatment abolished these effects of melatonin on Nrf2 as well as Sirt1 expression (Figures 8E–G).

Next, we measured HO-1 expression, a key anti-inflammatory agent regulated by Nrf2 (Li et al., 2018). Elevated HO-1 expression was detected in the melatonin-treated mice hippocampus (Figures 8A,D). However, this effect of melatonin was abolished after luzindole administration (Figures 8E,H), suggesting melatonin receptor-dependent regulation of HO-1 expression.

## Discussion

In the present study, we studied the neuroprotective effect of melatonin, against LPS-induced neuroinflammation and depressive-like behaviors. LPS treatment significantly induced depressive-like behaviors, oxidative stress, proinflammatory cytokines production, and NF-κB activation, followed by enhanced Iba-1 and GFAP expression in the hippocampus. However, the melatonin treatment attenuated depressive-like behaviors, oxidative stress, and neuroinflammation. Interestingly, luzindole treatment significantly abolished the effect of melatonin on depressive-like behaviors, oxidative stress as well as neuroinflammation. Moreover, melatonin mediated LPS effects on Sirt1/Nrf2/HO-1 signaling, which were abolished by luzindole treatment.

Lipopolysaccharide as cytokine inducer evoke peripheral and central immune activation in animal leading to neuroinflammation, accompanied by depressive-like behaviors (Sekio and Seki, 2015; Micheli et al., 2018; Zhao X. et al., 2019). In agreement with the previous findings (Raghavendra et al., 2000; Yu et al., 2017; Yuan et al., 2019), our results demonstrated depressive-like behaviors upon LPS-treatment, which was reversed by melatonin treatment. Besides, LPS dysregulates PI3K/Akt/GSk3b signalings, which plays a crucial role in survival, proliferation, and invasion by generating the second messengers (Guijarro-Munoz et al., 2014). Moreover, the PI3K-Akt/GSK3b pathway further regulates the induction and expression of inflammatory genes expression that contributes to the onset of depression. In our present study, decreased Akt/GSK3β phosphorylation was detected at 24 h post-LPS-treatment, which was significantly elevated by melatonin.

Growing evidence suggests that PI3K-Akt pathways regulate NF-κB expression and its downstream regulator (Duman and Voleti, 2012; Hussain et al., 2012; Kitagishi et al., 2012; Manning and Toker, 2017). NF-κB plays an important role in the regulation of immunity and inflammation (O’Neill and Kaltschmidt, 1997; Mattson and Camandola, 2001; Mémet, 2006; Seldon et al., 2007; Kassan et al., 2013). Upon activation via upstream regulatory elements such as Toll-like receptor-4, NF-κB translocate to the nucleus as a transcription factor and regulates numerous inflammatory regulators including TNF-α expression. Furthermore, increased TNFα, IL-6, and IL-1β in the LPS treated mice suggest immune imbalance which was determined via pro and anti-inflammatory cytokines measurement. Besides the neuroinflammatory pathways, free radicals such as ROS play a key role in the neuroinflammation by regulating pro as well as anti-inflammatory signaling (Munhoz et al., 2008). Dysregulated redox-sensitive signalings contribute a significant role in an immune imbalance accompanied by depression (Hsieh and Yang, 2013; Weyand et al., 2018). In the current study, our results showed that LPS significantly accelerated free radical generation, which was attenuated by melatonin treatment. Moreover, excessive free radicals such as ROS can induce NF-κB activation, followed by multiple inflammatory gene expression (Sarada et al., 2008; Hsieh and Yang, 2013; Lugrin et al., 2014), supporting the hypothesis that neuroinflammation leads to depression. Consistent with the previous reports (Salminen et al., 2008; Luo and Zhang, 2016; Shah et al., 2017), our findings showed that a significant Sirt1 gene suppression in the LPS-treated mice brain, which was improved by melatonin treatment. However, melatonin effects were abolished by luzindole treatment. Previously, Sirt1 dependent Nrf2 expression has been reported, and after inhibition of Sirt1 via inhibitor, enhanced pro-inflammatory cytokines and as well as p-NF-κB expression were detected upon LPS administration, suggesting a sirt1 role in LPS induced neuroinflammation (Mendez-David et al., 2015; Santofimia-Castaño et al., 2015; Song et al., 2017; Shah et al., 2017; Ma et al., 2018; Wang et al., 2019; Merlo et al., 2020; Yi et al., 2020; Zhi W. et al., 2020).

NF-κB regulates Nrf2 transcription and activity, whose downregulation/abrogation leads to increase NF-κB activity and enhanced cytokine production (Kratsovnik et al., 2005; Kawai and Akira, 2007; Lugrin et al., 2014; Djordjevic et al., 2015). Herein, melatonin treatment significantly increased Nrf2 and anti-inflammatory protein HO-1 expression which was down-regulated in the presence of melatonin receptor (MT1/MT2) inhibitor, suggesting the melatonin in a receptor-dependent manner regulates NF-κB/Nrf2/HO-1 expression (Rushworth et al., 2005; Rehman et al., 2019; García et al., 2020). HO-1 contributes a significant role in modulating the inflammatory response. Many anti-inflammatory mediators have been demonstrated to enhance HO-1 expression, which subsequently inhibits inflammation (Lee and Chau, 2002; Lee et al., 2003). Recently it has been demonstrated that melatonin acts through the Nrf2 pathway and prevents the decline of antioxidant enzyme activities during brain pathological conditions (Moezi et al., 2011; Mendez-David et al., 2015; Herrera-Arozamena et al., 2020; Zhi W. et al., 2020), supporting our results that endogenous melatonin counterbalances the oxidative stress by boosting the body antioxidants system via its receptors. Our results are also in agreement with growing evidence that melatonin enhances HO-1 expression via NF-κB, p38 MAPK, and Nrf2 cascade signaling mechanism (Santofimia-Castaño et al., 2015; Yu et al., 2017; Shah et al., 2017; Ma et al., 2018; Zhao et al., 2018; Rehman et al., 2019; Xi et al., 2019; García et al., 2020; Hein et al., 2020; Zhou et al., 2020; Zhi W. et al., 2020).

## Conclusion

In conclusion, our study showed that LPS treatment stimulates pro-inflammatory cytokines production and induce oxidative stress imbalance followed by NF-κB activation, which leads to neuroinflammation along with depressive-like behaviors. Also, LPS-treatment significantly reduced Akt/GSK3b phosphorylation as well as Sirt1 expression. Melatonin acts as a neuroprotective agent abolished LPS effects on oxidative stress, NF-κB activation, redox-sensitive signaling, and depressive-like behaviors in a receptor-dependent manner. Further, these findings also proposed an indispensable relation of the antioxidative and anti-inflammatory activities of melatonin. Finally, the molecular changes underlying melatonin’s effects may provide potential therapeutic candidates for the treatment of neuroinflammation associated depression.

## Data Availability Statement

All datasets generated for this study are included in the article/supplementary material.

## Ethics Statement

All experimental procedures were carried out according to the protocols approved by the Institutional Animal Care and Use Committee of Peking University Shenzhen Graduate School.

## Author Contributions

TA and QH designed the study, wrote the manuscript, and performed the experiments and data analysis. WL, SR, and NU helped with experimental work. NU, FS, IM, and YJ helped with the manuscript. YL, ZT, and SL supported the study. ZT and SL are the corresponding authors, reviewed and approved the manuscript, and held all the responsibilities related to this manuscript. All authors reviewed and approved the manuscript.

## Conflict of Interest

The authors declare that the research was conducted in the absence of any commercial or financial relationships that could be construed as a potential conflict of interest.

## Abbreviations

AKT: Serine-threonine protein kinase
ANOVA: Analysis of variance
BDNF: Brain-derived neurotrophic factors
CNS: Central nervous system
ELISA: enzyme-linked immunosorbent assay
FST: Force swimming test
GFAP: Glial Fibrillary Acidic Protein
GSK3: Glycogen synthase kinase 3
HO-1: Heme Oxygenase 1
IBA1: Allograft inflammatory factor 1
IL-1 ^β^: Interleukin 1 beta
IL-6: Interleukin 6
LPS: Lipopolysaccharides
MAPK: Mitogen-Activated Protein Kinase
MDD: Major depressive disorder
MT1/2: Melatonin receptor 1/2
NF- κ B: Nuclear Factor Kappa B Subunit
Nrf2, Nuclear Factor: Erythroid 2 Like 2
PIK3: phosphatidylinositol 3-kinase
ROS: Reactive oxygen species
TBARs: Thiobarbituric Acid Reactive Substance
TLR4: Toll-like receptor 4
TNF- α: Tumor Necrosis Factor-alpha.
